# Anticipated Visits of Eminent Members of the Profession in Great Britain

**Published:** 1850-03

**Authors:** 


*Anticipated visits of Eminent members of the Profession in Great Britain.*—It is announced in the Boston Medical and Surgical Journal, that Marshall Hall, M. D., and Dr. Forbes, late Editor of the British and Foreign Medical Review, may be expected in this country during the present year. They will meet with a hearty welcome.

